# Propensity score-matched evaluation of palliative transurethral resection and holmium laser enucleation of the prostate for bladder outlet obstruction in patients with prostate cancer

**DOI:** 10.1038/s41391-024-00831-1

**Published:** 2024-04-10

**Authors:** Alexander Tamalunas, Patrick Keller, Melanie Schott, Leo Federico Stadelmeier, Marc Kidess, Michael Atzler, Benedikt Ebner, Martin Hennenberg, Christian G. Stief, Giuseppe Magistro

**Affiliations:** 1 Department of Urology, University Hospital, LMU Munich, Munich, Germany; 2 Department of Urology, Asklepios Westklinikum Hamburg, Hamburg, Germany

**Keywords:** Prostate cancer, Cancer therapy, Prostate cancer

## Abstract

**Background:**

While transurethral resection of the prostate (TURP) is the standard-of-care, Holmium laser enucleation of the prostate (HoLEP) is widely accepted as a size-independent method for surgical treatment of patients with lower urinary tract symptoms (LUTS) secondary to bladder outlet obstruction (BOO). However, in an ageing society an increasing number of patients presents with BOO due to locally advanced prostate cancer. There is currently no guidelines recommendation as to the enucleation or resection technique. Therefore, we compared intraoperative performance, postoperative outcomes, and safety for palliative (p)TURP and (p)HoLEP.

**Methods:**

We conducted a retrospective, propensity score-matched analysis of 1373 and 2705 men who underwent TURP or HoLEP for LUTS/BOO between 2014 and 2021, respectively. Patients were matched for age, prostate size and preoperative international prostate symptom score (IPSS). Patients were stratified by technique and groups were compared for perioperative parameters, safety, and functional outcomes.

**Results:**

While postoperative symptoms and urodynamic parameters improved irrespective of technique, we report significantly increased resection and enucleation times for palliative indication. For corresponding efficiency parameters, we observed a two-fold higher surgical performance (g/min) for both techniques in patients without prostate cancer. While adverse events were comparable between groups, we found a two-fold higher hemoglobin drop in palliative patients.

**Conclusions:**

Currently, there is no standard-of-care for patients with BOO and locally advanced prostate cancer. Our data show that both TURP and HoLEP offer adequate symptom improvement and comparable safety profiles. While HoLEP is feasible even in larger prostates, both procedures become more difficult in patients with prostate cancer. Taken together, this study covers an important gap in current literature, helping urological surgeons to make evidence-based decisions for the benefit of their patients.

## Introduction

With steadily rising incidence due to increasing life expectancy, prostate cancer (PC) has emerged as a prevalent malignancy in ageing societies [1]. While PC in its early stages often remains undetected, displaying no clear symptoms, symptomatic presentation typically indicates locally advanced PC. Local disease progression can result in bothersome lower urinary tract symptoms (LUTS) and bladder outlet obstruction (BOO), significantly impacting quality of life (QoL) [2, 3], with a significant proportion of patients suffering from acute urinary retention (AUR) in their last year of life [3, 4]. For these patients, the primary goals of treatment involve preventing additional severe complications and enhancing QoL. Thus, disease management strategies for patients with locally advanced PC should prioritize palliative and minimally invasive approaches. While endocrine therapy is typically the initial treatment option for AUR caused by locally advanced and metastatic PC, when curative interventions are not feasible, it may require several months before observing any potential improvement in spontaneous voiding functions [4]. Thus, palliative transurethral resection of the prostate (pTURP) has most commonly been performed as treatment to achieve immediate relief of BOO for patients with locally advanced prostate cancer [3, 5]. While the possibility of impaired tumor control of incidental prostate cancer after TURP has recently been refuted [6], holmium laser enucleation of the prostate (HoLEP) has gained acceptance as a suitable alternative to TURP due to several advantages. HoLEP is nowadays recommended by both American Urological Association (AUA) and European Association of Urology (EAU) guidelines on the management of non-neurogenic male LUTS as size-independent endoscopic treatment method [7–11]. With its favorable safety profile HoLEP has constantly challenged TURP as surgical reference method and has since become the reference laser procedure for LUTS secondary to BOO [8, 12–16]. Even though the advantages of TURP and HoLEP are self-evident, the steep learning curve of HoLEP limits this procedure to high-volume tertiary referral centers [17]. Despite the specific advantages of both TURP and HoLEP for LUTS/BOO, published data on their use in the known presence of locally advanced prostate cancer and concomitant LUTS are scarce, and show worse outcomes than in a non-palliative setting [3, 5, 18]. However, those studies include either a small patient cohort, or have not been matched by preoperative patient data, while other studies emphasize the challenging aspects of performing these procedures in patients with PC [5, 19, 20].

In an ageing society with increasing numbers of patients presenting with LUTS/BOO due to locally advanced prostate cancer, guidelines recommendation on the use of either technique is urgently warranted. Therefore, we aim to address the feasibility of TURP and HoLEP and its potential benefits in patients who were under treatment due to biopsy-proven locally advanced prostate cancer. We retrospectively analyzed patients receiving TURP or HoLEP for LUTS/BOO in propensity score-matched cohorts, comparing each procedure in the palliative and non-palliative setting. We hypothesize that both procedures offer an adequate safety profile with immediate symptom control in the palliative setting compared to patients without PC.

## Methods

### Patient population and study design

To compare patients undergoing TURP or HoLEP for LUTS/BOO due to locally advanced prostate cancer, we performed a propensity score-matched analysis of a total of 1373 and 2705 men who underwent TURP or HoLEP for LUTS/BOO between 2014 and 2021, respectively. PC patients were offered TURP or HoLEP in addition to guideline-conforming PC treatment, and all procedures were indicated in accordance with the current EAU guidelines [21, 22] i.e., HoLEP procedures were reserved for patients with prostate size >50 cc, while TURP was indicated in patients with prostate sizes ≤50 cc. A computerized database was created for analyzing perioperative parameters, early functional outcomes, and safety profile for each procedure and each setting. Propensity score matching (PSM) was performed, and patients were matched 1:1 for the preoperative parameters prostate size, age, and preoperative symptom severity, measured with the international prostate symptom score (IPSS). Matching tolerance was set at 0.001, yielding a total of 180 propensity score-matched patients for TURP and HoLEP. Patients were stratified into groups by procedure and according to the presence of biopsy-proven locally advanced prostate cancer i.e., palliative (p)TURP and palliative (p)HoLEP. Thus, yielding four patient cohorts i.e., patients who underwent TURP for LUTS/BOO without PC, which were compared to patients who underwent TURP for LUTS/BOO due to locally advanced PC (TURP vs. pTURP), and patients who underwent HoLEP for LUTS/BOO without PC, who were compared to patients who underwent HoLEP for LUTS/BOO due to locally advanced PC (HoLEP vs. pHoLEP. Only patients in whom all the relevant information could be obtained, were included in the final analysis. Clinical and pathological information as well as perioperative data were used to describe the patient cohorts. Perioperative complications were analyzed in all groups and defined according to the modified Clavien-Dindo scale as any adverse event within 30 days of surgery [23].

All HoLEPs were performed by one experienced surgeon only, in a one-lobe enucleation technique, using VersaPulse^®^ 100 W Holmium Laser (Lumenis Ltd., Yokneam, Israel) with a frequency of 53 Hz and a power setting of 1.2 kJ. Morcellation was performed using a mechanical tissue morcellator (R. Wolf, Piranha, Knittlingen, Germany). All TURPs were conducted in bipolar technique using a 26F continuous flow resectoscope by three experienced surgeons, one of whom also performing the HoLEP procedures. According to our standard protocol a 24F three-way foley catheter was inserted after surgery and followed by 12 h of continuous bladder irrigation with normal saline. To rule out any learning curve bias, only HoLEPs performed by one experienced surgeon were included. The experienced surgeons performing TURP had each performed at least 100 procedures before their data was entered into the computerized database, ruling out any learning curve bias. All data were collected retrospectively.

### Statistical analysis

Statistical analysis and propensity score matching were performed using SPSS V29.0 software (IBM SPSS Statistics, Version 29.0. Armonk, NY), Preoperative, perioperative, and postoperative data are given as median and interquartile range (IQR) for continuous variables and as percentage for categorial variables. Normal distribution of variables was determined with Shapiro-Wilk test. Univariate analyses were performed using Fisher’s exact test and T test for categorical variables, and Mann-Whitney U test for continuous variables. For efficiency analysis, comparison of whole groups (TURP vs. pTURP, and HoLEP vs. pHoLEP) was performed by two-way analysis of variance. All reported p-values were two-sided and considered statistically significant if *p* < 0.05.

## Results

### Patient characteristics

Demographic parameters are displayed in Table 1A for patients who underwent TURP, and in Table 1B for patients who underwent pHoLEP, respectively. In total, we included 84 patients in the TURP group and 96 patients in the HoLEP group and stratified them according to indicated procedure (*n* = 42 TURP vs. *n* = 42 pTURP and *n* = 48 HoLEP vs. *n* = 48 pHoLEP). As propensity score matching was performed, there was no difference for prostate volume, age, and preoperative IPSS between groups. We report a median age of 71 years, a median preoperative IPSS of 25, and median prostate volume of 40 cc and 80 cc, for patients who underwent TURP or HoLEP, respectively. Furthermore, we report no statistically significant difference between groups regarding quality of life (QoL), maximum flow rate (Q_max_), post void residual (PVR), preoperative hemoglobin level (Hb), American Association of Anesthesiologists (ASA) score, or percentage of patients presenting with an indwelling urinary catheter (IDC). As anticipated total prostate specific antigen (PSA) and PSA density was significantly higher in palliative TURP groups. For patients who underwent TURP and pTURP, we report a median PSA of 3.5 ng/ml vs. 15.6 ng/ml and a median PSA density of 0.06 ng/ml/cc vs. 0.39 ng/ml/cc, respectively. There was no difference for patients who underwent HoLEP and pHoLEP, respectively.

**Table 1:** (**A**) Baseline characteristics TURP patients, (**B**) Baseline characteristics HoLEP patients.

A			
Characteristics			
	TURP (*n* = 42)	pTURP (*n* = 42)	*p* value
Age (yr)			
Median	71	71	0.871
IQR	69–77	69–77	
IPSS			
Median	25	25	0.528
IQR	21–28	21–29	
QoL			
Median	4	4	0.524
IQR	4–4.5	4–5	
PV (cc)			
Median	41	41	0.731
IQR	35–45	40–44	
Total PSA (ng/ml)			
Median	3.5	15.6	**0.008**
IQR	1.6–5.8	3.6–27.7	
PSA density (ng/mL/cc)			
Median	0.06	0.39	**0.01**
IQR	0.04–0.14	0.09–0.69	
Qmax (ml/s)			
Median	9	11	0.181
IQR	7–10.3	9–13	
PVR (ml)			
Median	182	155	0.325
IQR	164–196	91–310	
Hb (g/dl)			
Median	14.9	14.1	0.541
IQR	13.3–15.7	13.4–14.9	
ASA (%)			
≥3 vs < 3	45.2%	47.6%	0.827
IDC (%) *n*	47.6% (20)	52.3% (22)	0.586
Gleason score, n (%)			
≤6	–	1 (2.4%)	
7	–	1 (2.4%)	
8–10	–	40 (95.2%)	

B			
Characteristics			
	HoLEP (*n* = 48)	pHoLEP (*n* = 48)	*p* value
Age (yr)			
Median	76	76	0.78
IQR	73–79	72–80	
IPSS			
Median	24	24	0.743
IQR	20–27	22–26	
QoL			
Median	4	4	0.362
IQR	3.5–4.3	4–4	
PV (cc)			
Median	80	80	0.782
IQR	70–105	63–100	
Total PSA (ng/ml)			
Median	8.6	15.2	0.178
IQR	5.7–18.7	13.2–18.9	
PSA density (ng/mL/cc)			
Median	0.11	0.19	0.215
IQR	0.07–0.23	0.17–0.24	
Qmax (ml/s)			
Median	10	8	0.576
IQR	7.5–14	8.0–8.8	
PVR (ml)			
Median	80	101	0.522
IQR	4–162	64–126	
Hb (g/dl)			
Median	14.7	14.2	0.454
IQR	14.2–15.9	13.7–14.7	
ASA (%)			
≥3 vs < 3	54.2%	50.0%	0.838
IDC (%) *n*	37.5% (18)	45.8% (22)	0.535
Gleason score, *n* (%)			
≤6	–	2 (4.2%)	
7	–	16 (33.3%)	
8–10	–	30 (62.5%)	

*IQR* interquartile range, *IPSS* International Prostate Symptom Index, *QoL* quality of life, *PV* prostate volume, *PSA* prostate-specific-antigen, *Qmax* peak urinary flow rate, *PVR* postvoid residual urine, *Hb* haemoglobin, *ASA* American Society of Anaesthesiologists, *IDC* indwelling urinary catheter.

Bold values indicate statistically significant *p* values (*p* < 0.05).

### Perioperative assessment and functional outcomes

Table 2A and B show the analysis of perioperative data and short-term postoperative outcomes four weeks after surgery for patients who underwent TURP and HoLEP procedures, respectively. Considering postoperative functional parameters as well as QoL measurement differences, results for TURP vs. pTURP and HoLEP vs. pHoLEP were not statistically significant for IPSS, QoL, Q_max_, and for PVR. While all postoperative functional parameters improved in both groups four weeks after surgery, we found statistically significant differences in intraoperative efficiency parameters, favoring the TURP and HoLEP over pTURP and pHoLEP, respectively. There was no difference in catheterization time or length of hospital stay. Hemoglobin was assessed once preoperatively, and 24 h after surgery. While there was no statistical difference in postoperative hemoglobin drop in the TURP group, we report a statistically significant – albeit clinically irrelevant – difference in the overall median hemoglobin drop between HoLEP and pHoLEP groups.

**Table 2:** (**A**) Perioperative and postoperative outcome parameters TURP patients, (**B**) Perioperative and postoperative outcome parameters HoLEP patients.

A			
Clinical Outcomes			
	TURP (*n* = 42)	pTURP (*n* = 42)	*p* value
Δ IPSS			
Median	12	10	0.610
IQR	5–17	4–16	
Δ QoL			
Median	3	3	0.175
IQR	2–3	2–3	
Resected tissue (g)			
Median	26	21	0.292
IQR	15–37	18–24	
Hb drop (g/dl)			
Median	0.8	1.2	0.725
IQR	0.3–1.5	0.8–1.9	
Δ Qmax (ml/s)			
Median	13	14	0.382
IQR	11–14	11–16	
Δ PVR (ml)			
Median	105	110	0.138
IQR	83–203	90–156	
surgery time			
Median	51	67	**0.033**
IQR	38–66	50–104	
Efficacy (g/min)			
Median	0.6	0.3	**<0.001**
IQR	0.5–0.8	0.1–0.5	
Catheterization time (d)			
Median	2	2	0.275
IQR	2–3	2–2	
Hospitalisation time (d)			
Median	3	3	0.245
IQR	3–4.8	3–5	

B			
Clinical Outcomes			
	HoLEP (*n* = 48)	pHoLEP (*n* = 48)	*p* value
Δ IPSS			
Median	15	12	0.765
IQR	8–22	7–19	
Δ QoL			
Median	3	2	0.136
IQR	2–3	1–4	
Resected tissue (g)			
Median	61	65	0.073
IQR	49–72	50–70	
Hb drop (g/dl)			
Median	0.7	1.3	**0.007**
IQR	0.1–1.3	0.8–1.9	
Δ Qmax (ml/s)			
Median	19	12	0.122
IQR	8–30	4–19	
Δ PVR (ml)			
Median	61	52	0.166
IQR	10–111	12–94	
Enucleation time			
Median	19	35	**<0.001**
IQR	12–26	31–40	
Morcellation time			
Median	9	16	0.222
IQR	3–15	12–19	
Efficacy (g/min)			
Median	3.2	1.8	**0.001**
IQR	2.8–3.6	1.6–2.1	
Catheterization time (d)			
Median	2	2	0.225
IQR	2–3	2–2	
Hospitalisation time (d)			
Median	3	3	
IQR	3–4	3–3	0.275

*IQR* interquartile range, *IPSS* International Prostate Symptom Index, *QoL* quality of life, *Qmax* peak urinary flow rate, *PVR* postvoid residual urine, *Hb* haemoglobin.

Bold values indicate statistically significant *p* values (*p* < 0.05).

### Perioperative complications

Overall, we observed 11 adverse events (AEs) in the TURP group (11/84, 13.1%), and 10 AEs in the HoLEP group (10/96, 10.4%), shown in Table 3A and B, resepctively. For describing and grading complications, the modified Clavien-Dindo score was used. Overall, 4 patients receiving TURP and 7 patients with pTURP, and 4 patients with HoLEP and 6 patients with pHoLEP had at least one perioperative complication, which did not yield a statistically significant difference. We divided complications into minor (Clavien I) and major complications (Clavien II to V), requiring an intervention. While the complication rate was a little higher in the palliative groups, this did not yield statistical significance. Complications and respective management are listed in detail in Table 3 (A and B, respectively).

**Table 3:** (**A**) Treatment related adverse events (AEs) according to the Clavien-Dindo classification TURP patients, (**B**) Treatment related adverse events (AEs) according to the Clavien-Dindo classification HoLEP patients.

A			
Adverse events (AEs)			
	TURP (*n* = 42)	*p*TURP (*n* = 42)	*p* value
Overall AEs; *n* (%)	**4** (9.5%)	**7** (16.7%)	0.521
Clavien Dindo I	**0**	**0**	
Urinary retention			
Clavien Dindo II	**1** (2.4%)	**2** (4.8%)	1.0
Clot retention	1	1	
Clavien Dindo III	**3** (7.1%)	**5** (11.9%)	0.713
Urinary retention	0	1	
Clot retention	2	1	
Bleeding	1	3	
**B**			
**Adverse events (AEs)**			
	**HoLEP (*n*** = **48)**	**pHoLEP (*n*** = **48)**	***p*** **value**
Overall AEs; *n* (%)	**4** (8.3%)	**6** (12.5%)	0.74
Clavien Dindo I	**0**	**0**	
Urinary retention			
Clavien Dindo II	**0**	**1** (2.1%)	1.0
Clot retention	0	1	
Clavien Dindo III	**4** (8.3%)	**5** (10.4%)	1.0
Urinary retention	1	1	
Clot retention	2	1	
Bleeding	1	2	
Urethral stricture	0	1	

Bold values indicate statistically significant *p* values (*p* < 0.05)

## Discussion

While prostate cancer is common in patients >65 years of age, median survival after diagnosis can reach several years to even decades, and patients with PC usually live with tumor for several years after diagnosis [1, 6]. Complications such as hematuria, BOO, urinary retention, bladder stone, and hydronephrosis are common in men with end-stage PC, and up to 20% of advanced PC patients suffer from acute urinary retention (AUR) in their last year of life [24, 25]. Even though patients can choose radiotherapy and androgen blockade, most demonstrate a strong desire to quickly relieve LUTS and improve their QoL. Current approaches include taking α_1_-blocker monotherapy, intermittent catheterization, suprapubic cystostomy, and pTURP. While α_1_-blocker monotherapy comes with class-specific adverse events, including postural hypotension, relief of symptoms in patients suffering from BOO due to concomitant PC may be limited. However, long-term intermittent catheterization or suprapubic cystostomy are not capable of significantly improving QoL and are not acceptable for patients. Thus, pTURP for BOO secondary to PC has been adopted by urologists for several decades [5]. However, and to the best of our knowledge, data on treatment of patients suffering from LUTS/BOO and concomitant PC is still scarce [3, 5, 18]. Thus, our patients were offered TURP or HoLEP in addition to guideline-conforming PC treatment [22]. Current guidelines aim to evaluate and recommend different treatment options for LUTS/BOO, and in case of an obstructive prostate recommendation is mainly based on prostate size, and treatment modality (TURP vs HoLEP vs OP) [7]. Since its introduction, HoLEP has shown to be a size-independent method with efficacy and safety even outranking TURP and OP [8, 10, 13, 26]. With recent introductions of different HoLEP techniques, such as the en-bloc enucleation technique, HoLEP challenges OP, especially for larger prostates [15]. However, the increasing number of patients suffering from LUTS/BOO and concomitant prostate cancer prompted us to investigate the feasibility of TURP and HoLEP and its potential benefits in patients with biopsy-proven locally advanced prostate cancer in large propensity score-matched cohorts.

While HoLEP was previously shown to be feasible in patients suffering from BOO secondary to PC [18], there is no current study matching patients for age, prostate size and preoperative IPSS, comparing intraoperative performance, complications, and postoperative outcomes. Primarily, our results show that TURP and HoLEP are both feasible procedures in patients with LUTS/BOO secondary to PC. This is in line with the current, scarce literature [3, 5, 18]. The intraoperative course in both our patient cohorts was uneventful, and a low rate of postoperative complication was observed, not exceeding those so far reported for standard HoLEP [8, 13, 26].

As patients were propensity score-matched, we found no difference in age, IPSS, or prostate volume in the pTURP vs TURP and the HoLEP vs pHoLEP cohort. However, PV was almost two-fold in the HoLEP cohorts. Although the age difference between HoLEP and TURP cohorts was not clinically significant, this may reflect the age-dependency of prostate size observed by various studies before [8, 27]. As this was a retrospective analysis, the aim was not to compare the two procedures, rather patients were selected for HoLEP or TURP based on prostate size, according to the current guidelines on the management of male LUTS, and later compared [21]. Additionally, we found no difference in preoperative Q_max_, observed unsatisfying QoL scores of 4 points throughout our patient cohorts, as well as a clinically relevant and equally dissatisfying PVR, without any difference between groups. As anticipated, we observed a significantly higher median PSA in patients with prostate cancer, with concomitant difference in PSA density between groups. However, with larger prostates in the HoLEP cohorts, this difference was not statistically significant, supporting the hypothesis that PSA also correlates with prostate size. Thus, confirming to our previous data [28].

While we observed no significant difference in patients presenting with an ASA score ≥III between groups, with equal numbers of patients presenting with an IDUC prior to surgery in the palliative groups [24, 25]. With >95%, we found significant prostate cancers (Gleason grade ≥7) throughout our PC cohorts. Only few patients had Gleason grade 6 prostate cancers, making them eligible for AS. However, locally advanced, histologically more aggressive tumors may account for LUTS/BOO secondary to PC, corresponding to tumor burden [3, 5].

While there was no significant difference in preoperative hemoglobin value, there was a difference in 24-h postoperative hemoglobin drop between the palliative cohort and the BPH cohort, albeit statistically significant only for the HoLEP procedure. However, there was no need for perioperative blood transfusion, corresponding to the data we gathered on the favorable perioperative safety profile of performing HoLEP in octogenarians [29]. However, angiogenesis is part of the neoplastic process in prostate cancer, and our data reflect the increased challenge of endoscopic LUTS surgery in patients suffering from BOO secondary to PC. To minimize adverse events, many surgeons prefer to perform a channel TURP in patients with PC and LUTS [5]. However, we did not perform channel TURP in PC patients, as evidenced by the amount of resected tissue, with no difference between PC patients and BPH patients. For our HoLEP cohort median hemoglobin drop did not differ from the corresponding TURP or pTURP patients, even though the prostates were twice larger. As prostate hyperplasic growth may be accepted as neoplastic process, involving angiogenesis, our HoLEP data are much more favorable [30]. Laser enucleation of larger glands results in a larger surface of the prostatic fossa, consequently influencing the amount of fluid absorption during HoLEP and increasing the risk of hemodilution over operating time [8, 17, 31]. Thus, hemodilution may well explain the increased – albeit clinically insignificant – blood loss in patients with enlarged prostates.

We report significantly prolonged surgery and enucleation time, for TURP and HoLEP procedures, respectively, comparing the BPH with the PC patient cohorts. This is corroborated by the corresponding efficacy (g/min) values, favoring BPH patients. In TURP procedures this may reflect the increased difficulty operating on PC patients, with markedly more vulnerable prostatic tissue. In HoLEP patients the surgical plain is obviously more challenging to follow in cancerous areas. However, morcellation time did not significantly differ in PC patients, following HoLEP. Considering the one-lobe technique not only circumvents the laborious protocol of repeatedly finding the surgical plane, as once the correct plain is entered, the adenoma is mobilized in an en-bloc fashion, it also prevents the obvious time lost between finding and morcellating three separate lobes versus one single lobe, especially in vulnerable PC tissue [15].

Although surgical efficacy parameters did not significantly differ between our patient cohorts (BPH vs PC), all patients in our study showed improvement of functional outcomes after TURP or HoLEP. There were distinct improvements of IPSS, Q_max_, and PVR for all patients in our study with similar improvement throughout groups. There was no statistical difference favoring the BPH patient cohort, making both procedures eligible in PC patients. However, we observed markedly increased postoperative functional parameters in the HoLEP groups, highlighting the clinical benefits of laser enucleation, especially considering the twice enlarged prostates in the HoLEP cohorts. These findings correspond with international literature, and our previous results [8, 13, 15, 26]. Furthermore, QoL improved similarly for all patient cohorts.

Overall, 21 patients suffered a postoperative complication according to the modified CDC. Most of our complications were found to be CDC grade III, with persistent hematuria or clot retention, requiring surgical reintervention as the most common grade III complications. The rate of grade III complications (11.9% for pTURP and 10.4% for pHoLEP) did not significantly outrank those in the BPH cohort, and seems favorable, compared to other reports for pTURP [5, 32], pHoLEP data [3, 18], with postoperative voiding failure in up to 42%, need of repeated TURP in up to 29%, and permanent incontinence in up to 10% [5, 32]. Even though complications were more common in PC patients, there was no statistically significant difference to BPH patients, regarding overall or higher grade (≥II CDC) complications. Even though the risk of hemodilution due to a significantly larger surface of the prostatic fossa in larger glands may be higher, one of the many advantages of HoLEP and bipolar TURP includes using physiologic saline as irrigant. Thus, we found no life-threatening transurethral resection (TUR-) syndrome in our patient cohort. Thus, we report no CDC grade IV or grade V complications among our patient cohort.

Limitations of our study surely include its retrospective design and short post-operative follow-up period. Thus, limiting the power of our conclusion. Following up the patient at a tertiary referral center is problematic, preventing complete collection of data for more cases. However, a longer follow-up is required for complete appraisal of functional outcomes and the safety profile. However, Elshal and colleagues could show no significant difference in short-term (30 days) postoperative functional outcomes compared to follow-up after one year [33]. Taken together, we could show that there are no limitations to using TURP or HoLEP even in patients with PC.

Currently, there is no standard-of-care for patients with LUTS/BOO and locally advanced prostate cancer. However, our results show that TURP and HoLEP are both feasible procedures in patients with LUTS/BOO secondary to PC. HoLEP procedures were performed in patient cohorts with twice larger prostate size, showing that it is a feasible technique in a palliative setting in patients with prostate sizes, in which TURP may no longer be recommended. However, both procedures offer adequate and fast symptom relief with comparable safety profiles. and corresponding efficiency parameters resulting in two-fold higher surgical performance for both techniques in patients without prostate cancer. Taken together, the study covers an important gap in current literature, helping urological surgeons to make evidence-based decisions for the benefit of their patients. Thus, future research should focus on a multicenter approach to facilitate a guidelines recommendation for patients suffering from BOO due to locally advanced prostate cancer.

## Data Availability

The data that support the findings of this study are available from the corresponding author upon reasonable request.
